# Study Protocol for the Development of an African Framework for Critical Care Nursing Based on Ubuntu Using Participatory Cooperative Inquiry: Decolonising Care

**DOI:** 10.1111/nicc.70456

**Published:** 2026-03-30

**Authors:** Chris Carter, Michael Kanyanta, Joy Notter

**Affiliations:** ^1^ Birmingham City University Birmingham UK; ^2^ University of Zambia Lusaka Zambia

## Abstract

**Background:**

Critical care nursing in Zambia is a new speciality that has adopted traditional nursing documentation and currently uses the Roper, Logan and Tierney's Activities of Daily Living combined with NANDA International (NANDA‐I) criteria. It is a cause for concern that these models are based on different high‐income domains and are not critical care or context‐specific, making integration and application in practice challenging. Therefore, there is an urgent need to develop a Zambian context‐specific critical care nursing framework.

**Aims:**

This study aims to explore intensive care nurses' perceptions of the applicability of currently used nursing models and theories and to develop a new Zambian critical care nursing framework based on the principles of Ubuntu.

**Methods:**

This study will employ a two‐phase, mixed‐methods approach using a participatory cooperative inquiry design. Phase 1 includes a retrospective review of nursing care plan documentation. This will allow for the identification of the current use of nursing models and theories. Phase 1 utilises documentary data analysis to identify key nursing documentation. Descriptive statistics and, where possible, significant difference will be used to determine patterns and trends. Phase 2 will utilise the findings from phase 1 as the basis for semi‐structured focus groups with intensive care nurses within the study site. Total population sampling will be used; therefore, all nurses will be invited to participate. Framework analysis will be used to analyse the qualitative data sets.

**Conclusion:**

The outcomes will illustrate the efficiency and effectiveness of nursing documentation and perceptions of using the current nursing model. The findings will be combined to develop a critical care nursing framework based on Ubuntu, which will then be piloted.

**Relevance to Clinical Practice:**

This study has been designed in response to recognition that critical care nursing practice needs to context specific to enhance the nursing care provided.

## Impact Statements

- What is known about the topic
  ○ Critical care provision in low‐ and low–middle‐income countries is increasing, with nursing care often based on models from a high‐income country.
  ○ There is increasing recognition for the need of context‐specific nursing models in this setting.
  ○ Ubuntu has been identified as an emerging model of nursing within the central, southern African region; however, its use in critical care is unknown.
- What this paper adds
  ○ This study provides a methodological framework to assess and evaluate the extent to which current nursing models are used in critical care practice.
  ○ The framework developed will provide an example for the application of a context‐specific African philosophy in the development of critical care nursing as an emerging speciality in Africa.
  ○ This study is one of the first context‐specific critical care nursing research projects to evaluate the current critical care nursing model to develop a new culturally appropriate critical care nursing framework.

## 1 Background

Globally, critical care nursing is advancing with the result that survival from critical illness is increasing; however, survival in low‐income countries remains limited [1]. Zambia is classified by the World Bank [2] as a low‐ to middle‐income country with a GINI Index of 51.5%. Over the past decade, critical care nursing has been gradually advancing with the introduction of specialist nurse education programmes from diploma to master's level [3]. These changes are essential as critical care units are increasingly providing complex, high‐intensity, time‐specific care, with patients fully dependent on the expertise of the workforce. Key elements of good nursing care are the assessment, planning and implementation of care; for these to be effective, it is essential that they are specifically designed for use within the health system delivering the care. Clinical outcomes depend on rapid, effective responses to changes in patient status. Therefore staff systems that includes documentation that can be easily completed, is context‐specific and is applicable for the setting in which the unit is based [4, 5]. It is therefore an ongoing cause for concern that the current national model of critical care nursing in Zambia is based on the Roper, Logan and Tierney's Activities of Daily Living [6] and the NANDA International (NANDA‐I) (formerly, the North American Nursing Diagnosis Association) diagnosis criteria [7]. These two models were designed for two different, but high‐income countries and, in consequence, it is challenging to integrate them into any system, based on different philosophical assumptions and assessing different domains (Box 1). The situation is compounded because, as Box 1 illustrates, neither are critical care–specific nor designed for resource‐limited settings, and address different domains. To add to these technical differences, in no way do either of them reflect African philosophies of living and society [8]. Thus, when aiming to develop context‐specific critical care nursing care, nurses in Zambia are faced with a plethora of challenges when trying to apply these different principles to their practice [9, 10]. It is therefore perhaps not surprising that although the theory of these models is taught in student nurse education programmes and post‐qualifying higher‐level qualifications, there appears to be little application into practice. In addition, although Zambia has retained a focus on these nursing models, the limited published evidence on their use in resource‐limited settings, further demonstrating their lack of applicability.

**BOX 1:** Similarities and differences between Roper, Logan and Tierney's Activities of Daily Living, NANDA‐I and Ubuntu.

	Activities of daily living	NANDA‐I	Ubuntu
Underpinning concepts	Person as an individual with functional needs.	Person as a responder to health problems	Person in relation to family, community and society
	Designed for Western settings	Designed for US settings	African philosophy and can be adapted to African nursing context
	Explicitly holistic about independence and focuses on physical, psychological, social and environmental	Holistic if the nurse selects diagnoses based on health problem	Holistic at a human and social level.
	Used to guide nursing care	Standardised nursing diagnoses	Guides nursing values and relationships with patients and families.
	Activity‐based framework	Taxonomy of diagnoses	Philosophical and ethical
	A person with varying levels of independence	A set of actual or potential human responses	A human being embedded in family, culture and community.
Use in critical care	Patients are often fully dependent. Not systems focused does not fit well with critical illness. Could be useful in critical care recovery.	Works well with protocols, documentation and multi‐disciplinary teams, which are challenging in resource constrained settings. Task centred and based on diagnoses.	Humanises critical care. Cultural and context‐specific ethical decision‐making structure with patient and family central. Built around a moral and ethical compass.

As Tierney [11, 12] has pointed out, nursing models did have their place in nursing. However, globally their use has continued to decline, with specialist nurses and new fields of nursing focusing on developing patient pathways for specific diseases and conditions [13]. This alternative approach is supported by Vieira et al.'s [14] critical review of the literature, which found that many previously accepted critical care models are outdated with few studies supporting their implementation, resulting in them remaining theoretical elements of education and training. In consequence, when discussing the challenges with stakeholders, they were clear that they wanted a Zambian context‐specific conceptual framework which could be operationalised within the current healthcare system. Carter's [8] Zambian study found that specialist critical care nurses in Zambia are deeply concerned that the current model of nursing is outdated and only seen as a theoretical part of their education. In practice, it is not fully understood by nurses (from student to MSc level) and has limited connection with critical care nursing or the African context. Therefore, it could be argued that for critical care nurses in Zambia to advance the nursing agenda, there is an urgent need to challenge the philosophies and courses that have been implemented in education and practice [15]. Thus, although it is accepted that Ubuntu philosophy was also not designed for critical care, nevertheless by definition its main concepts fit more nearly with Zambian familial and social patterns, making adaptation for application in intensive care possible.

When identifying possible philosophical frameworks, searches revealed the increasing recognition for Ubuntu across the African continent as offering an appropriate construct to build upon [16, 17, 18]. As Mugumbate and Chereni [19] point out, the key translation of ‘I am because we are’ typifies the way of life, the shared consciousness and interconnectedness that exist in African societies. The concepts inherent in this approach are therefore part of shared societal attitudes and would translate well into a context‐specific framework for critical care nursing service delivery. This would straddle both nursing strategies and patient and family needs and expectations, enabling the clinical care to be efficient, effective and culturally sound.

## 2 Aims

The interlinked aims of this study are to explore the knowledge and understanding critical care nurses have of the current nursing models officially, identifying positives, challenges and constraints. These will then be used to underpin the conceptualisation and development of a new context‐specific framework for critical care nursing based on the identified African philosophy of Ubuntu, and of direct applicability in Zambia.

## 3 Design and Methods

**FIGURE 1:**
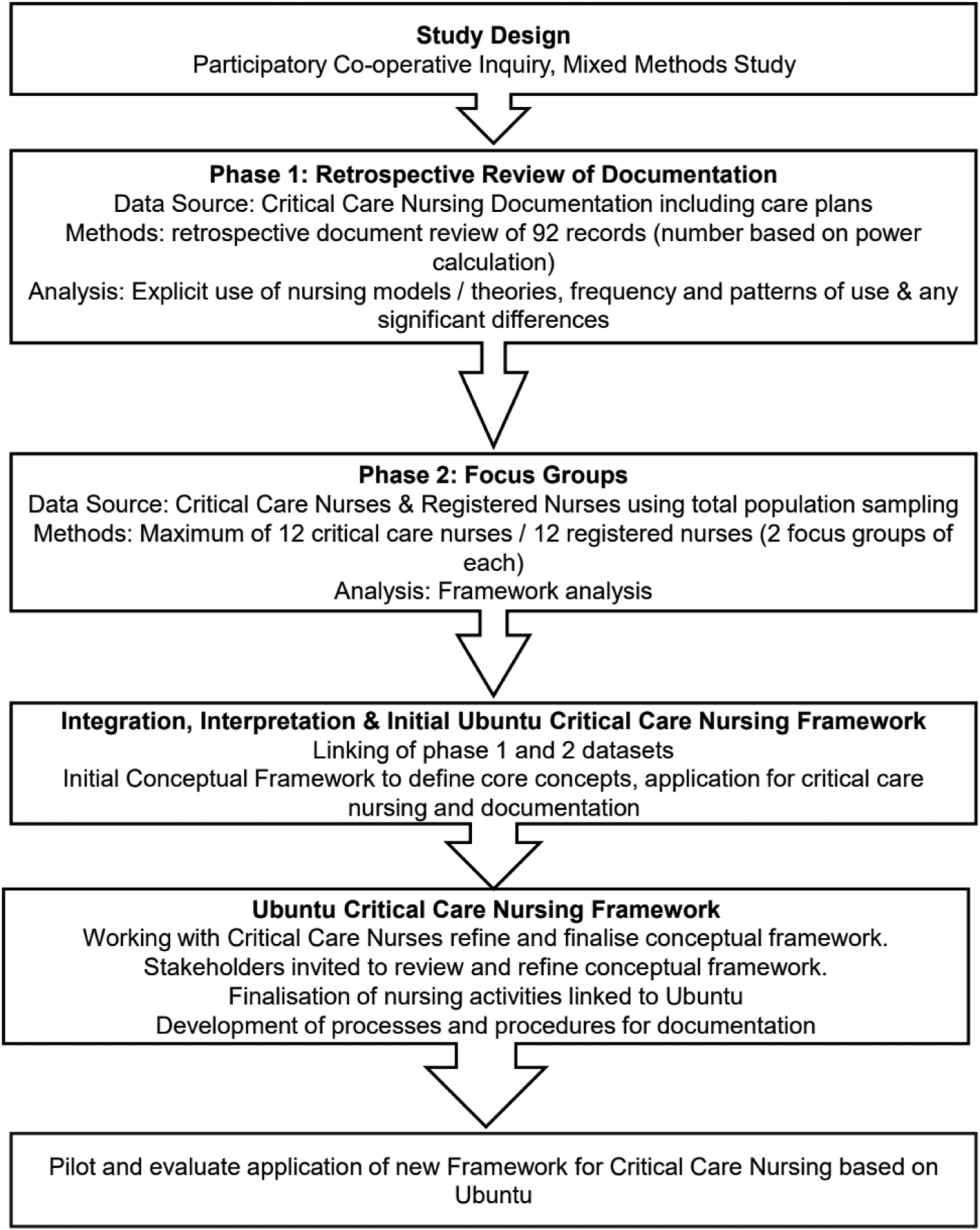
Schematic overview of the proposed research phases and development of the framework.

In line with the principle of Ubuntu, the study will use participatory cooperative inquiry throughout [20], as this approach with its shared ownership and focus on empowerment through shared multi‐directional learning is seen as essential. The aim is to empower critical care nurses and ultimately the Ministry of Health such that on the completion of the study, they take ownership of the findings of the study. This could then be translated for other specialist groups and nursing education programmes and could help align nursing care to the Zambian society. The study will have two phases; all steps of data collection and analysis will follow the guidelines for the method chosen, thereby re‐enforcing the context specificity, appropriateness and acceptability of processes used and all outcomes [21]. Figure 1 provides a schematic overview of the proposed research phases and development of the framework.

Phase 1: Documentary data collection and analysis to compile a retrospective review of nursing care plans [22]. This will allow for the identification of the types of nursing problems identified, and on the use of the Roper, Logan and Tierny and NANDA‐I diagnosis, nursing plans and evaluation.

Phase 2: A series of focus groups with critical care nurses working in the intensive care unit (ICU). This will include both registered nurses and registered critical care nurses. Framework analysis will be used in this second phase as a core aspect of this approach to data collection and analysis is context checking, which will be conducted by co‐researchers' [23].

### 3.1 Setting and Sample

This study will be conducted in one ICU in a national referral hospital in Zambia.

The ICU consists of 10 beds with the ability to provide invasive ventilation, and an additional 10 high‐dependency beds. This ICU provides service to a population of 21 million people, and as a result, the unit admits a wide variety of specialties for both adults and children. While the limitations of a single‐centre study are acknowledged, this facility serves as the primary ICU for the entire country. Therefore, findings will be shared with stakeholders, including the Ministry of Health, the Nursing and Midwifery Council of Zambia (NMCZ), professional organisations such as the Critical Care Nurses Association for Zambia (CCNAZ), ICU leadership teams and hospital management who will participate in all developments for application in practice. They can then lead post‐study projects for transferability to other, smaller ICUs.

#### 3.1.1 Participants

Data for Phase 1 will be collected over a 1‐month period in April 2026. Access has been sought and gained (see the Ethics section) to review nursing documentation in patient records. The main ICU consists of 10 beds; accordingly, all patient care plans over a 4‐week period will be audited. A review of admissions and discharge statistics reveals that on average 45 patients a month are admitted. A power calculation for a simple proportion using $n=\frac{Z^{2}P\left(1-P\right)}{d^{2}}$ was carried out to identify the number of patient care plan records to audit [24]. In the calculation, a confidence interval of 95% was used with a 5% degree of error. Z‐score was calculated on the basis of a previous pilot study, which was completed to identify the proportion of records containing evidence of use of the current models. This calculation suggested that 92 records should be audited using a pre‐designed extraction framework. During Phase 1, strategies to recruit participants for the focus groups will be implemented. As a resource‐limited setting there are few critical care nurses with mainly registered nurses working in ICU, and in consequence total population sampling will be used. This includes 10 registered critical care nurses. Therefore, all registered critical care nurses will be invited to participate, with a similar number of registered nurses selected by randomisation from those who volunteer. The ages of potential participants range from 24 to 55, with males comprising approximately one‐third of the total cohort.

#### 3.1.2 Inclusion and Exclusion Criteria

Inclusion criteria for the retrospective care plan review include invasively ventilated, critically ill patients admitted to ICU, registered nurses and registered critical care nurses working in the main ICU. Exclusion criteria include non‐critically ill patients, for example, post‐operative recovery patients requiring overnight recovery, those admitted to the high‐dependency area and those that do not meet the requirement critical care as outlined by the World Federation of Societies of Intensive and Critical Care Medicine [25] and non‐registered nurses, for example, student nurses, medical staff and other professional groups, such as physiotherapists.

### 3.2 Data Collection Tools and Methods

All data will be collected by a Zambian researcher (MK) with support from the international team (CC and JN). A formalised document data extraction grid will be developed, piloted and peer‐reviewed for documentation audit (Table 1) and focus groups (Table 2).

**TABLE 1:** Documentation audit summary.

Criteria	Rationale
Demographic information and reason for admission.	To identify types of patients admitted and to identify potential nursing problems.
Review of actual written nursing records for terminology, type of nursing care plan and evaluation.	Each section of nursing documentation needs to be considered and compared.
Check whether NANDA‐I checklist is used. Formal NANDA‐I validated list comprising all items of the scale.	To review whether any, some or all criteria are utilised and recorded as part of decision‐making and care provided.
Check Roper, Logan and Tierney's checklist is used. Formal validated list comprising all items of the scale.	To review whether any, some or all criteria are utilised and recorded as part of decision‐making and care provided.
Any other approach/system used identify and create chart for recording.	To review whether any other system of nursing documentation is used as part of decision‐making and care provided.
Standards of nursing documentation.	To identify whether documentation of best practice standards are maintained. To check that documentation is contemporaneous.

**TABLE 2:** Focus group summary.

Criteria	Rationale
Demographic information.	Information about participants professional career, for example, length of time working in the ICU, type of nurses involved in study and participant numbers.
Semi‐structured interview schedule of eight to nine questions with prompts.	To facilitate discussions and enable key points to be raised—in line with framework analysis guidance.

### 3.3 Data Analysis

The retrospective review of nursing care plans will use the extraction framework as indicated in Table 1. Data will be collected and transferred into a MS Excel spreadsheet. Data will be numerical: for example, how many times a NANDA‐I diagnosis is used and how many times the ADLs are included. This approach was seen as a practical measure to assess actual usage of the current recommended models. Descriptive statistics will be used to determine patterns and trends [26]. Where possible, tests for significant difference such as chi‐squared will be used to determine significant differences, as the majority of the data will be nominal or ordinal [27]. For open‐ended questions, data will be coded and analysed using framework analysis processes [23].

Focus groups will be conducted with different nursing groups as outlined above. A maximum of six participants will be invited to each focus group, and MS Teams will be used to record the discussions, as this will facilitate immediate transcribing. Focus groups will be scheduled during the handover period to allow participants to attend, to reduce the impact on patient care and will be conducted in an office away from the clinical area, but within the hospital. Following the focus group, the MS Teams transcript will be downloaded and saved as an MS Word document. Framework analysis will then be used to analyse the interviews [23]. Only the research team will have access to the raw data sets. All data will be analysed by the research team, including a Zambian Researcher who will provide context validation for the coding process. Framework analysis is considered more transparent and robust than thematic analysis [28]. Open coding of the transcript entails constant comparisons supporting the emergence of initial categories. A more focussed second in‐depth analysis includes reviewing data for context [28, 29]. This iterative analysis facilitates interconnectedness [28, 30], facilitating analysis, both within and across data sets [29]. This will include searching for phrases that fit with the Ubuntu philosophical discussion.

### 3.4 Ethics and Research Approvals

To promote equity and in accordance with Zambian requirements, ethics approval to complete this study was obtained from the University of Zambia Biomedical Research Ethics Committee (Ref.: UNZA‐7130/2025), and the study registered with the National Health Research Authority (Ref.: NHRA‐2723/22/09/2025). Approval to conduct this study has been granted by the hospital management team and the Senior Medical Superintendent. In addition, as this study involves an international team, full ethics approval was granted by the Birmingham City University Ethics Committee (Ref.: 15192). There is a risk that poor practice may be identified during the study, for example, poor documentation and incorrect care planning. However, it is accepted that participant confidentiality and data protection are crucial; therefore, any concerns will be addressed in a sensitive manner to avoid demoralising staff and a training package will be delivered to all staff. In addition, good practice identified during the study will be highlighted and shared. Outcomes will be included as part of the dissemination. In addition, all participants are registered nurses who are required to complete ongoing professional development/continuing professional development (CPD) as part of their annual re‐registration with the NMCZ [31]. Therefore, the dissemination/training event will contribute to nurses' annual revalidation requirements.

## 4 Discussion

The urgent need to develop a context‐specific framework for critical care nursing in Zambia has been recognised and accepted by stakeholders and critical care nurses [8]. A key argument in this is that the HIC models of nursing are based on differing societal values, infrastructures and finances [12, 32, 33]. Adopting a framework based on Ubuntu recognises that caring, an essential component of nursing, cannot be viewed in isolation. In the African context, it involves recognising all aspects of an individual's being, which includes immediate and extended family members, ancestors, the community (and village) they come from and their tribe [34]. Zambia is not alone in seeking to contextualise care; within the African context, there is an increasing move to utilise African philosophies, such as Ubuntu as potential nursing models. These take account of the individual, the community and society as a whole. Adopting Ubuntu in critical care accepts the argument that the interconnectedness and shared societal responsibility extend to education and through that to practice [35].

Mulaudzi and Gundo [35] developed an Ubuntu community model in nursing in selected provinces in South Africa that demonstrated the interconnectedness of all aspects of life on health. Similarly, Carter [8] developed a career structure and conceptual framework for critical care nurses in Zambia based on the philosophy of Ubuntu. This recognises the individual and collective role of nurses and families, revealing how this can improve holistic care, and through that patient outcomes. Therefore, this study has the potential to build upon previous work and develop a framework for critical care nursing practice that will be fit for purpose for the country in which it will be used. There needs to be a focus on re‐designing processes and procedures to include placing the patient within their cultural and social place, rather than solely focusing on tasks resulting from domain diagnoses. This in turn facilitates individualised person‐centred care with documentation focused on the totality of care and the inclusion of context‐specific care designed to maximise the possibility of re‐integration into family and the community [15]. This contributes towards futureproofing the acceptability and appropriateness of critical care nursing practice. It will also fit with the move away from traditional colonial care practise that is evident across the African continent [36]. This will enable Zambia's critical care nurses to move forwards with their peers and develop unique care that is tailored to the population, following their own cultural heritage and patterns. In addition, this approach will also provide sustainability, build research capacity and empower local nurse researchers so that they can become independent. It is also important to note that research generated by local researchers and their recommendations may have a better uptake among African policy makers than research produced internationally as it is culturally and context‐specific [37, 38].

### 4.1 Development of the Framework

The overall approach framework used in this study is such that all activities entail interaction with key stakeholders. The statistical analysis will indicate the extent to which the current models are used in nursing documentation. This will also identify the differences to Ubuntu philosophy [35, 36]. Integration of the quantitative data sets and the findings from the focus groups will be used to facilitate review of the current documentation, and how well these fit with the care given. They will be used to support the development of the proposed new framework to provide excellence in critical care professional practice [39, 40]. As Mugumbate and Chereni [19] suggest, using Ubuntu principles will change the approach to care, to one in which the interconnectedness, shared responsibility and progression, link the care with service providers and the care given. Therefore, documentation needs to be couched in language that reflects the shared vested interest in the individual within the group [41]. Key issues will be extracted from the integrated data sets and essential points for the new framework developed with stakeholders [36].

## 5 Limitations

It is accepted that this is a small‐scale study in a single‐centre study; however, as the leading ICU in the country, findings may still be generalisable. In addition, this is one of the first studies in this area of critical care nursing practice and will provide insight into the current use of nursing models and provide an opportunity to develop an initial Ubuntu‐based framework for critical care nurses, which could then be scaled up. A review of the model by stakeholders will support refining and adjustments to the overall structure of the framework, an approach successfully used in a previous study to review and update specific documentation [42]. To reduce bias, researcher triangulation will be used during the data collection and analysis. All researchers will maintain a reflexive journal; this will be used throughout to check that research positionality does not adversely affect analysis. As this study involves an international research team, debriefing and supervision is an integral component as it will challenge assumptions and support the clarity needed to develop the new framework. Data processing will also be audited; this will be carried out by an independent researcher to further minimise bias.

## 6 Implications for Practice

Little has been published from low–middle‐income countries on context‐specific critical care nursing models and frameworks. However, as critical care nursing evolves and matures, it is important that context‐specific frameworks and models for practice are developed. Ubuntu has been identified as a possible context‐specific philosophy that can be translated for use in nursing care; however, it has not yet been applied to critical care nursing. Critical care in many low‐income countries is advancing, and this study is seen as a first step in the development of a context‐specific critical care nursing framework. Stakeholders (including the Ministry of Health and NMCZ) will play a key role in dissemination and implementation of the findings. They are keen to see processes developed that are culturally competent and will align practice to the needs of the population they serve. Also using participatory cooperative inquiry approach, future pilots of the new model will be led by stakeholders who have the research expertise to lead and guide such ventures.

## 7 Conclusion

This protocol provides an overview of a study to develop a context‐specific critical care nursing framework using an African philosophy to underpin practice. It offers an opportunity for critical care nurses to be empowered to lead and develop their own specialist area of practice.

## Author Contributions

C.C. conceptualised the study. J.N. and M.K. contributed equally to the development of this new study. All authors contributed to the preparation of this manuscript.

## Ethics Statement

This study has been approved by University of Zambia Biomedical Research Ethics Committee (Ref: UNZA‐7130/2025), Birmingham City University, UK (Ref: 15192), and registered with the National Health Research Authority (Ref: 272322/09/2025).

## Consent

The authors have nothing to report.

## Conflicts of Interest

The authors declare no conflicts of interest.

## Data Availability

The data that support the findings of this study are available from the corresponding author upon reasonable request.
